# Investigating the Effect of Esterification on Retinal Pigment Epithelial Uptake Using Rhodamine B Derivatives

**DOI:** 10.1167/tvst.9.6.18

**Published:** 2020-05-19

**Authors:** Chandima Bulumulla, Ruvanthi N. Kularatne, Timothy Catchpole, Alison Takacs, Abigail Christie, Alexa Gilfoyle, Timothy D. Nguyen, Mihaela C. Stefan, Karl G. Csaky

**Affiliations:** 1 Retina Foundation of the Southwest, Dallas, TX, USA; 2 Department of Chemistry and Biochemistry, The University of Texas at Dallas, Richardson, TX, USA; 3 Department of Bioengineering, The University of Texas at Dallas, Richardson, TX, USA

**Keywords:** cellular penetration, fluorescent probes, mitochondria, age-related macular degeneration

## Abstract

**Purpose:**

This study investigated the effects of esterification and increased lipophilicity on cellular penetration, accumulation and retention in ARPE-19-nic cells using ester functionalized rhodamine B dyes.

**Methods:**

Rhodamine B was esterified to generate four dyes with increasing lipophilicity. Cellular uptake, retention and mitochondrial localization were investigated in vitro using ARPE-19-nic cells using direct intracellular and extracellular and mitochondrial fluorescence quantitation, confocal and high-resolution live cell imaging and co-localization with Mito-GFP.

**Results:**

Cellular penetrance, mitochondrial accumulation, and retention of the esterified dyes were increased in ARPE-19-nic cells compared with the nonesterified parent dye by direct fluorescence quantitation. Imaging demonstrated intracellular accumulation was confined to mitochondria as confirmed by colocalization with Mito-GFP.

**Conclusions:**

Esterification is an effective way to increase lipophilicity of a dye to improve cellular penetration of chemical entities. These observations may be key to improving retinal drug delivery for retinal pigment epithelium–based diseases.

**Translational Relevance:**

Understanding the intracellular distribution of drugs into retinal pigment epithelium cells is a critical component for identifying potential therapies for retinal pigment epithelium–based diseases.

## Introduction

Drug delivery into the posterior segment of the eye poses a major challenge owing to the unique anatomic and physiologic barriers.^1^ There are three main barriers present in the eye: (i) static barriers, including the sclera, conjunctiva, and retina, along with blood–aqueous and blood–retinal barriers; (ii) dynamic barriers, including choroidal and conjunctival blood flow, lymphatic clearance, and tear dilution; and (iii) P-glycoprotein pumps and other water efflux mechanisms.^1^^,^^2^ To achieve high in vivo efficacy, drugs need to effectively move through multiple membranes before reaching the target of interest.

Cellular penetrance of an active pharmaceutical ingredient can be enhanced by making more lipophilic versions or prodrugs,^3^ attachment of the active pharmaceutical ingredient to an active transporter,^4^^,^^5^ chemically conjugate the active pharmaceutical ingredients into organic polymers via pendant groups,^6^ or with aid of subcellular organelle-directed molecular cages.^7^^,^^8^ Of these techniques, the prodrug approach is the most straightforward, because it requires the least alteration of the active pharmaceutical ingredient. Altering the lipophilicity of a prodrug can be critical, because drug transport across membranes may depend on the lipophilicity index.^9^^–^^11^ Once prodrugs enter the cells, they are metabolized either chemically (depending on the pH) or enzymatically (esterase, ketone reductase, steroid 6β-hydroxylase, etc.) to yield the unmodified therapeutic agent.

One of the key questions in ocular drug delivery to the retinal pigment epithelium (RPE) is the extent to which lipophilicity and an ideal charge is required to deliver drug candidates into the RPE. To address the need, drugs can be modified in a precise manner to control the lipophilicity. Compared with drugs, fluorescent dyes can be easily detected and can be used as a model system to track and quantify amounts in cellular and tissue levels. In addition, new insights can be gained on clearance mechanisms, the effect of lipophilicity, charge, and the effect of chemical functional groups toward drug delivery.

With recent advancements in disease pathology, mitochondria are known to be important in a wide range of diseases including cancer,^12^ diabetes,^13^ cardiovascular disease, and age-related neurodegenerative diseases.^14^^,^^15^ Age-related macular degeneration (AMD) is the leading cause of blindness in persons over the age of 55 years in developed countries. Supported by AMD donor tissue experiments,^16^^–^^18^ there is an emerging hypothesis that mitochondria in RPE cells are a primary site of AMD pathology. Therefore, drugs that can penetrate the RPE and specifically target mitochondria are of paramount importance.

To elucidate the structure–property relationship in chemical constructs, we synthesized four dyes with different degrees of lipophilicities and evaluated their cellular penetration, accumulation, and retention abilities in RPE cells. Mitochondrial uptake was identified by both organelle isolation and co-localization with mitochondrially targeted green fluorescent protein.

## Methods

All commercial chemicals purchased from Sigma Aldrich (St Louis, MO) and Fisher Scientific (Waltham, MA) were used without further purification unless otherwise noted. ^1^H NMR spectra were recorded on a 500 MHz Bruker AVANCE III spectrometer using deuterated chloroform as the solvent. Multiplicities were given as: s (singlet), d (doublet), t (triplet), q (quartet), or m (multiplet). Optical characterizations were carried out using Dulbecco's phosphate-buffered saline (DPBS) solutions with equal concentrations of all the dyes. UV-vis spectra were recorded on a Thermo Scientific NanoDrop 2000 spectrophotometer with 2048-element linear silicon Charge-Coupled Device (CCD) array and fluorescence spectra were obtained from a SpectraMax Gemini EM microplate reader connected to a photomultiplier tube detector at λ_ex_/ λ_em_ = 552 nm/558 nm. Confocal laser scanning microscope images were recorded on a Carl Zeiss LSM 800 system (Carl Zeiss Meditec, Jena, Germany) equipped with an Axio Observer microscope and images were analyzed using Zen 2.5 (blue edition) software. Reverse phase high-performance liquid chromatography was performed on an Agilent (Santa Clara, CA) 1100 Series HPLC equipped with an Agilent autosampler (model G1313A) and an Agilent FLD detector (model G1321A)

### Synthesis of the Dyes

General synthesis route for esterification reactions: to a 100-mL single-neck, round-bottom flask was added rhodamine B (0.2 g) with 20.0 mL of relevant alcohol. To the stirring solution, concentrated HCl (2.0 mL) was added dropwise and refluxed overnight. After cooling the reaction mixture to room temperature, excess alcohol was removed under reduced pressure. The pure compound was isolated by using column chromatography with silica as the stationary phase. Two successive column purifications were performed, first with 100% ethyl acetate as the eluent and second with isocratic elution using ethyl acetate and methanol (for rhodamine B methyl ester and rhodamine B ethyl ester = 3:2 *v/v* and rhodamine B propyl ester and rhodamine B butyl ester = 4:1 *v/v*). All the rhodamine B esters were obtained as dark purple solids. To carry out cellular uptake and imaging experiments, 1 mM stock solutions were made in sterilized DMSO and stored in aliquots at −20°C.

*Rhodamine B Methyl Ester*: Yield = 96%, ^1^H NMR (500 MHz, CDCl_3_); δ 1.33 (t, *J* = 7.0 Hz, 12H), 3.65 (qt, *J* = 7.0 Hz, 8H), 3.68 (s, 3H), 6.83 (d, *J* = 2.0 Hz, 2H), 6.93 (dd, *J* = 4.7 & 2.5 Hz, 2H), 7.06 (d, *J* = 4.7 Hz, 2H), 7.32 (d, *J* = 7.0 Hz, 1H), 7.75 (m, 1H), 7.82 (m, 1H), 8.29 (d, 7.5 Hz, 1H)

*Rhodamine B Ethyl Ester*: Yield = 90%, ^1^H NMR (500 MHz, CDCl_3_); δ 1.10 (t, *J* = 7.0 Hz, 3H), 1.35 (t, *J* = 7.0 Hz, 12H), 3.70 (qt, *J* = 7.0 Hz, 8H), 4.10 (qt, *J* = 7.0 Hz, 2H), 6.88 (s, 2H), 6.93 (d, *J* = 9.5 Hz, 2H), 7.10 (d, *J* = 9.5 Hz, 2H), 7.34 (d, 6.8 Hz, 1H), 7.77 (m, 1H), 7.83 (m,1H), 8.29 (d, 8.0 Hz, 1H)

*Rhodamine B Propyl Ester*: Yield = 75%, ^1^H NMR (500 MHz, CDCl_3_); δ 0.78 (t, *J* = 7.0 Hz, 3H), 1.32 (t, *J* = 7.0 Hz, 12H), 1.47 (dd, *J* = 7.0 & 14.0 Hz, 2H), 3.64 (qt, *J* = 7.0 Hz, 8H), 3.97 (t, *J* = 7.0 Hz, 2H), 6.81 (d, *J* = 2.0 Hz, 2H), 6.91 (dd, *J* = 2.5 & 9.5 Hz, 2H), 7.07 (d, *J* = 9.5 Hz, 2H), 7.30 (d, *J* = 7.5 Hz, 1H), 7.74 (m, 1H), 7.78 (m, 1H), 8.29 (d, *J* = 8.0 Hz, 1H)

*Rhodamine B Butyl Ester* (RBBE): Yield = 70%, ^1^H NMR (500 MHz, CDCl_3_); δ 0.79 (t, *J* = 7.0 Hz, 3H), 1.16 (m, 2H), 1.32 (t, *J* = 7.0 Hz, 12H), 1.39 (m, 2H), 3.64 (qt, *J* = 7.5 Hz, 8H), 4.00 (t, *J* = 6.5 Hz, 2H), 6.81 (d, *J* = 2.5 Hz, 2H), 6.90 (dd, *J* = 2.0 & 9.5 Hz, 2H), 7.07 (d, *J* = 9.5 Hz, 2H), 7.30 (d, *J* = 7.5 Hz, 1H), 7.73 (m, 1H), 7.78 (m,1H), 8.27 (d, *J* = 8.0 Hz, 1H)

### Cell Culture Experiments with ARPE-19-nic Cells

ARPE-19 cells were cultured in T-75 flasks using DMEM: F-12 (ATCC 30-2006) supplemented with 10% fetal bovine serum (FBS) until 95% to 100% confluency was reached. Subsequently, cells were harvested and seeded in 6-well plates according to a published procedure using MEM-Nic media.^19^ For all the experiments, the cells were used at passages 25 to 30. All cell culture reagents were purchased from Invitrogen-Gibco (Grand Island, NY) and ATCC (Manassas, VA).

### Stability of Dyes in Media and in ARPE-19-nic Cells

Fresh 100 nM RBBE solutions were prepared in MEM-Nic + 1% FBS with 0.2% Tween20. HPLC chromatograms were recorded immediately after making solutions and after incubation at 37°C for 2 hours to determine stability of the ester linkage in media.

For cell work, confluent ARPE-19-nic cells were treated with 100 nM dye solutions for 30 minutes and 2 hours at 37°C, 5% CO_2_ in a 12-well plate. After washing the cells with PBS three times, cells were extracted using cell scrappers with the aid of methanol. The cell suspension in methanol was sonicated in a water bath for 5 minutes and was centrifuged at 16,000 *g* for 15 minutes. The supernatant was transferred to an HPLC vial and methanol was evaporated under a stream of nitrogen. The sample was resuspended in 40 µL of methanol before injecting into the HPLC system. Twenty microliters of the samples were injected with an autosampler. Separation was conducted by 1.1 mL/min isocratic elution with a methanol (80%) and 10 mM sodium phosphate buffer (pH 5.8, 20%) mobile phase on a 250 × 4.6-mm (5 µm) C18 column (Pinnacle II; Restek, Bellefonte, PA) maintained at 40°C. The samples were monitored at an emission wavelength of 580 nm (λ_ex_ = 550 nm) using a FLD detector and analyzed with Agilent Chemstation software.

### Quantification Experiments

Black clear bottomed Costar 96-well plates were pretreated with 0.039 mg/mL collagen I solution prepared in sterilized deionized water (50 µL per well) for 1 hour, aspirated and washed with 100 µL of DPBS. Confluent ARPE-19-nic cells were harvested and seeded at approximately 70,000 cells per well and grown until the cells reached 100% confluency. At the time of confluence (day 0), media were removed from the cells and treated with 100 µL of freshly made dye solutions in MEM-Nic + 1% FBS in amber Eppendorf tubes in dark. After the appropriate incubation time, cells were washed thrice with DPBS, lysed with Qiagen RLT buffer with 0.2% Tween20, and fluorescence was recorded at λ_ex_/ λ_em_ = 552 nm/558 nm. To determine intracellular and extracellular dye levels, cells were treated with 25 nM dye solutions for 2 hours. Extracellular levels were measured by transferring 75 µL of the media sitting on top of the cells to a new black, clear bottomed 96-well plate. To the transferred media, 25 µL of Qiagen RLT lysis buffer with 0.2% Tween20 were added and fluorescence was measured as mentioned elsewhere in this article. Intracellular amounts were determined by adding 100 µL of Qiagen RLT lysis buffer with 0.2% Tween20 to the cells that were washed thrice with DPBS. Fluorescence measurements were carried as described. For accumulation experiments, freshly prepared 25 nM dye solutions were added to cells multiple times (1×, 2×, or 3×) and incubated for 2 hours after each addition, removing media before each addition. After the incubation period, cells were washed three times with DPBS and fluorescence was measured from a plate reader. For retention experiments, cells were treated with 25 nM dye solutions for 2 hours at 37°C, 5% CO_2_, and fresh media was added every 24 hours after the removal of the dye solution.

### Co-Localization Experiments with Mito-GFP

ARPE-19-nic cells were seeded at 1.5 × 10^5^ cells per well density in 8-well Millicell EZ slides with MEM-Nic + 1% FBS. After 24 hours (60%–70% confluency), cells were transfected with CellLight Mitochondria-GFP using manufacturer's recommended protocol. For co-localization experiments carried out at subconfluent conditions, media containing CellLight Mito-GFP were removed, washed with DPBS and cells were treated with 25 nM dye solutions prepared in MEM-Nic + 1% FBS at 37°C, 5% CO_2_. After 30 minutes, media containing dye solution was removed and cells were washed three times with DPBS. Cells were mounted with DPBS for confocal laser scanning microscope imaging. An 63× oil lens was used to image cells in green and red channels. For experiments carried out at confluent conditions, transfection was carried out at 60% to 70% confluency and cells were grown to 100% confluency. A 20× lens was used to generate images to compare staining efficiencies.

To analyze RBBE and its association with mitochondria, high-resolution live cell imaging was conducted using a Zeiss LSM880 inverted confocal microscope equipped with a GaAsP detector. All the images were obtained by using a 63× oil objective (30°C oil) in three channels (blue, green, and red) at room temperature. ARPE-19-nic cells were grown in Lab-Tek II chambered no 1.5 German coverslip system for 24 hours before treating with CellLight Mito-GFP overnight followed by a 30-minute treatment of 25 nM RBBE in growth media. After treating the cells with RBBE, cells were washed twice with PBS and nuclei were stained employing a Hoechst stain (1/2000 diluted from a 10 mg/mL stock solution in media) for 10 minutes at room temperature. Subsequently cells were washed twice with PBS and cells were imaged in phenol red free CO_2_ independent Leibovitz's (1×) media.

### Quantification of Dye Amounts in Isolated Mitochondria

ARPE-19-nic cells were grown in 60 cm^2^ dishes in MEM-Nic + 1% FBS to until the cells were 100% confluent. Ten milliliters of 100 nM Dye solutions (RB/RBBE) prepared in growth media were added to the cells and incubated for 30 minutes. After removing media-containing dyes, cells were washed with warm HBSS (10 mL) and 3 mL of 0.25% Trypsin-EDTA was added and incubated at 37°C, 5% CO_2_ for 10 minutes. Then, 7 mL of growth media was added and cells were extracted to a 15 mL centrifuge tube. After centrifuging the cell suspension at 300 rcf for 5 minutes, supernatant was removed and cell pellet was resuspended in 2 mL of isolation buffer (0.25 M sucrose and 10 mM HEPES). Cells were disrupted using a probe sonicator (Misonix S-3000) for 10 seconds in ice. Subsequently, intact cells and debris were removed by centrifuging at 1000*g* for 10 minutes. Supernatant was collected, partitioned into two, and centrifuged at 20,000*g* for 25 minutes. Pellet containing mitochondria were saved and washed using 0.5 mL of isolation buffer. After centrifuging at 20,000*g* for 25 minutes, one-half of the mitochondrial pellet was used to quantify dye amounts using RLT lysis buffer (Qiagen) with 0.2% Tween20 and the other half was used to extract DNA using the QIAamp DNA mini kit (Qiagen). To confirm the mitochondria isolation protocol using PCR, primers were sourced from a previously published report.^20^ Primers AAG TTC GCA TGT CCT AGC ACC and TGA CGC AAA GCA CAT AAA GTC C target a 213-bp section of the Beta-2 Microglobulin gene (nuclear target), and primers GCC ACA GCA CTT AAA CAC ATC TCT and TAG GAT GGG CGG GGG T target a 186-bp sequence between nucleotides 322 to 508 of the mitochondrial genome. PCR using these primers were performed on DNA from the mitochondrial fraction and compared with total DNA samples from ARPE-19-nic cells. PCR conditions were: 5 minutes at 95°C denaturation, followed by 23 cycles of 30 seconds at 95°C, 15 seconds at 60°C, 10 seconds at 72°C, and finally a 4°C hold. PCR products were quantified using the Quant-iT Picogreen dsDNA Assay kit (Invitrogen), and ratios of mitochondrial to nuclear amplification were calculated for each sample.

## Results

### Synthesis and Characterization of Rhodamine B Derivatives

**Figure 1.**
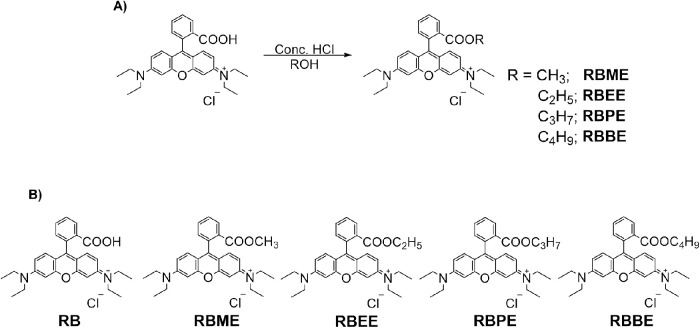
(A) Chemical synthesis of the dyes, (B) structures of RB and RB ester derivatives.

Synthesis of the rhodamine B derivatives were carried out via a Fischer esterification procedure as shown in Figure 1A. All the ester derivatives were obtained in satisfactory yields after column purifications. Proton NMR characteristics agreed with previously reported data (Supplementary Figs. S1 to S4).^21^ Because the objective of the experiment was to study both esterification and increased lipophilicity, carbon chain length of the ester unit was incrementally increased without changing the fluorophore skeleton (Fig. 1B). Optical characteristics of the dye molecules were tested using UV-vis spectroscopy and fluorescence spectroscopy (Supplementary Fig. S5). All the dyes have similar absorption profiles (λ_max_ ∼ 550 nm), although RB demonstrated higher fluorescence values compared with that of the ester derivatives.

### Stability, Cellular Uptake, Accumulation and Retention Ability of the Dyes in ARPE-19-nic Cells

**Figure 2.**
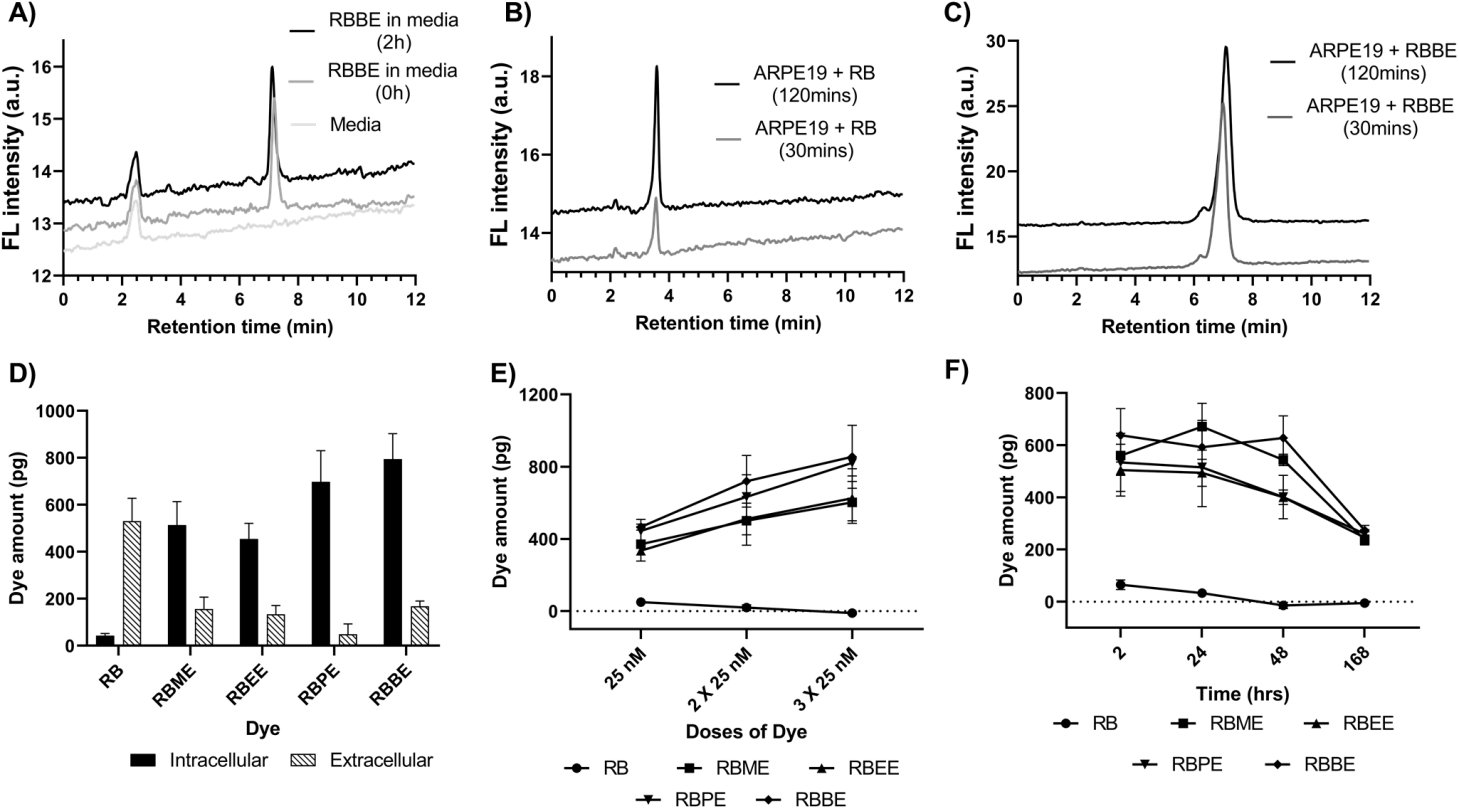
(A) HPLC chromatogram of 100 nM RBBE in MEM-Nic + 1% FBS after 0 and 2 hours. (B) HPLC chromatogram of cell extracts treated with 100 nM RB at 30 and 120 minutes. (C) HPLC chromatogram of cell extracts treated with 100 nM RBBE at 30 and 120 minutes, (a.u. = arbitrary units). (D) Fluorescence readings comparing intracellular and extracellular levels in ARPE-19-nic cells at 2 hours after dye exposure. (E) Dye amounts in ARPE-19-nic cells after multiple doses of RB and its ester derivatives. (F) Dye amounts in ARPE-19-nic cells after a single dose of RB and its ester derivatives at different time intervals.

To ensure ester forms are stable under in vitro experimental conditions, stability of RBBE (most lipophilic RB ester) was evaluated using HPLC for 2 hours in MEM-Nic media supplemented with 1% FBS (Fig. 2A). Because the ester dye was stable in media up to 2 hours, we selected 2 hours as our maximum time point for further experiments. Moreover, we treated confluent ARPE-19-nic cells with 100 nM RB and RBBE for 30 minutes and 2 hours and extracted dyes from cells to qualitatively assess cellular penetration and the de-esterification processes taking place inside the cells (Figs. 2B and 2C). As shown in Figure 2C, RBBE did not convert into its parent form within 2 hours, allowing us to quantitate fluorescence readings using standard curves for accumulation and retention experiments. The dose dependency was studied by treating ARPE-19-nic cells with 10, 25, and 50 nM concentrations in media for 2 hours at 37°C, 5% CO_2_ (Supplementary Fig. S7). At a dose of 10 nM, there was no significant difference in fluorescence between RB and the ester derivatives. At 25 nM, a difference was observed between RB and all of the ester forms (2-way analysis of variance [ANOVA]; *P* < 0.0001). Because the 25 nM dye concentrations did not show any cytotoxicity (Supplementary Fig. S6) or saturation effects (Supplementary Fig. S7), this concentration was selected for next steps. The cell permeability of ester-derived dyes was further confirmed by quantitating the amounts of dyes in intracellular and extracellular environments (Fig. 2D). The intracellular levels of all esterified versions of the dyes were higher than the parent dye RB (2-way ANOVA; *P* < 0.001). For all dyes, the intracellular and extracellular levels were different (*P* < 0.001); however, RB was primarily located in the extracellular space while the esterified versions were located intracellularly. Next, the accumulation ability was investigated by treating the cells repeatedly with dyes at the same concentration every 2 hours (Fig. 2E). The intracellular levels of the ester functionalized dyes increased with multiple doses (2-way ANOVA; *P* < 0.05); however, levels of the RB parent dye showed no accumulation. The retention ability of the dyes was tested by first treating the cells with dyes for 2 hours followed by incubation with fresh media without dyes. For 2- and 7-day trials, media was replaced every 24 hours. Retention profiles for the dyes were obtained by measuring the intracellular fluorescence intensities (Fig. 2F). After 48 hours, levels of the esterified dyes did not significantly change from the levels detected at 2 hours (2-way ANOVA). At 1 week after exposure, the esterified dyes were still detectable in the RPE cells.

### Selective Dye Accumulation in Mitochondria

**Figure 3.**
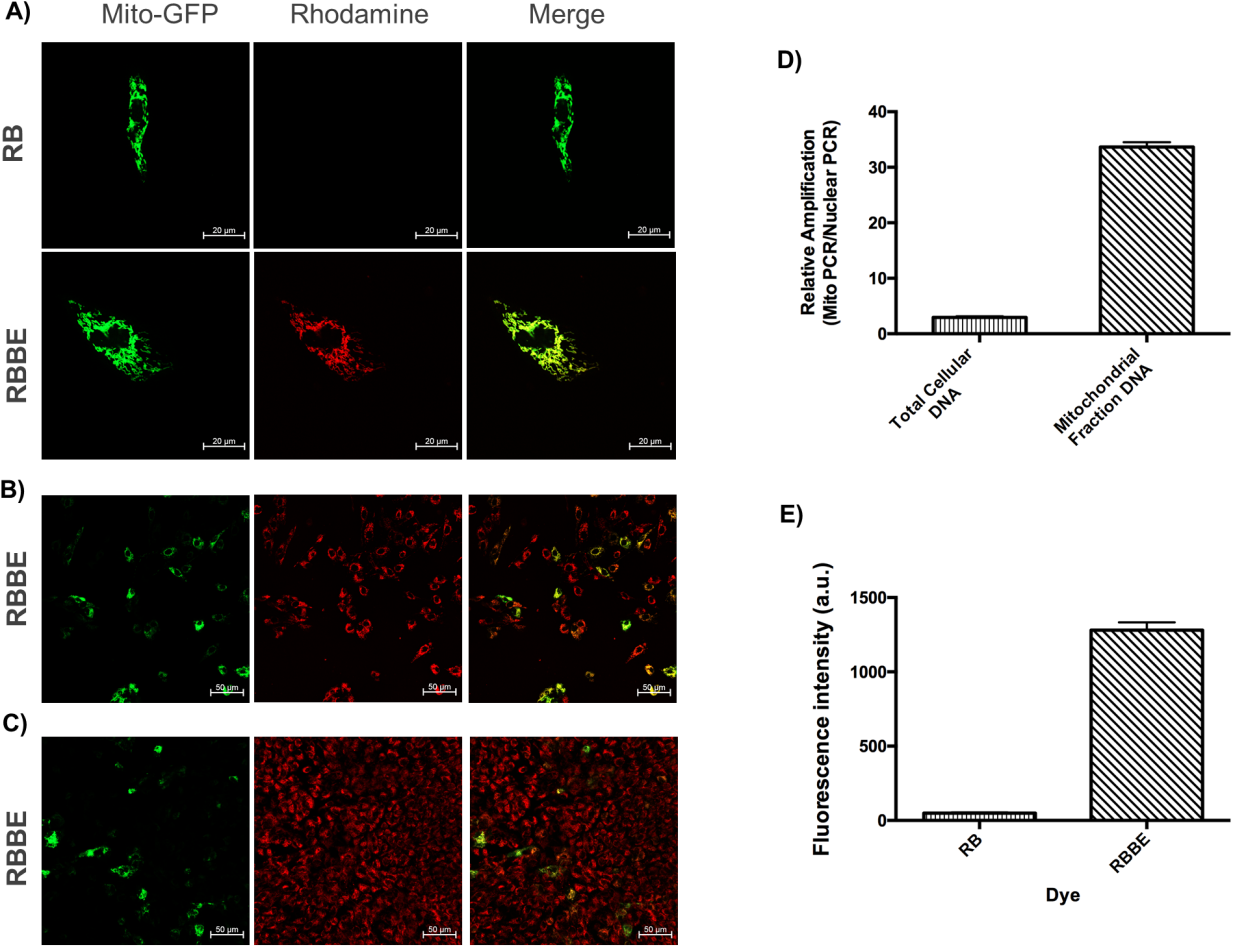
(A) Co-localization experiment carried out for RB and RBBE at 25 nM (scale bar = 20 µm). (B) Mitochondrial staining ability of RBBE for nonconfluent ARPE-19-nic cells and (C) confluent ARPE-19-nic cells (scale bar = 50 µm). (D) Ratio of MitoPCR/NuclearPCR for total (genomic + mitochondrial) and mitochondrial DNA fractions. (E) Fluorescence readings on extracted mitochondria after treating ARPE-19-nic cells with RB and RBBE.

The dye molecules presented here have a positively charged nitrogen atom. It was theorized that the esterified dyes may accumulate in the mitochondria based on the inherent negative mitochondrial inner membrane potential.^22^ To validate the mitochondrial targeting ability of these dyes, they were subjected to co-localization experiments with CellLite Mitochondria-GFP BacMam 2.0, a viral construct that labels mitochondria with GFP. As shown in Figure 3A, RB was not detected within the cells. The ester derivatives co-localized with the mitochondria, as shown in Figure 3A and Supplementary Figure S8. Compared with Mito-GFP, all the ester derived dyes evenly stained the mitochondria irrespective of the confluency (Fig. 3B). As further confirmation, dye uptake was studied in isolated mitochondria. Mitochondria were extracted according to a previously published protocol.^23^ Confirmation of mitochondrial isolation can be seen in Figure 3C. After isolation, the ratio of mitochondrial to nuclear DNA was significantly increased (*t*-test; *P* = 0.0103). Figure 3D demonstrates increased fluorescence activity from isolated mitochondria for cells treated with RBBE compared with cells treated with RB (*t*-test; *P* < 0.0001).

**Figure 4.**
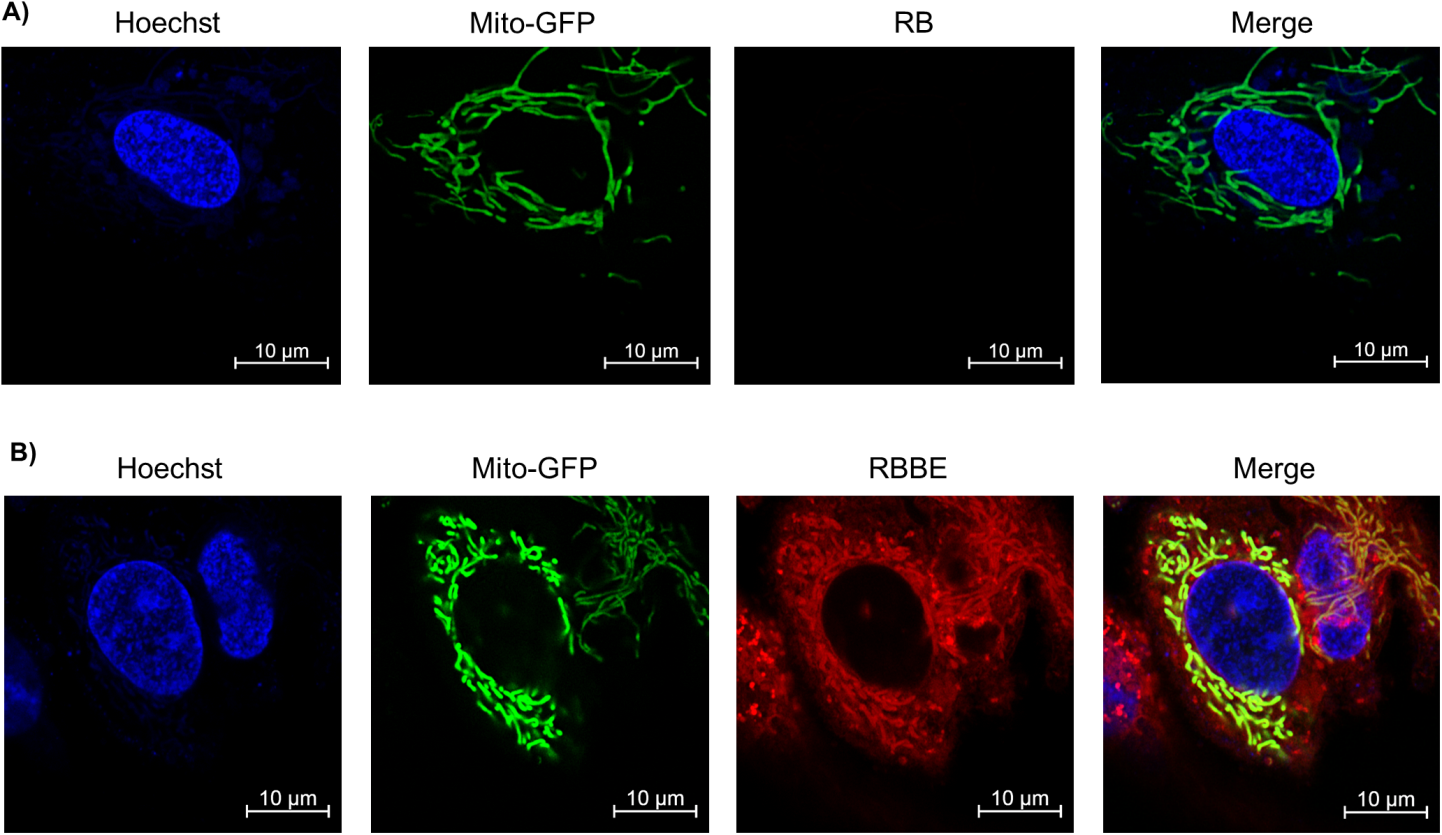
Airy scan confocal microscopy images of ARPE-19-nic cells treated with RB (A) and RBBE (B).

To further confirm RBBE's association with mitochondria, high resolution confocal microscopy images were obtained under low laser powers (<0.2) and later enhanced using deconvoluted airy scan processing. Because very little laser power was employed no photobleaching was observed. As shown in Figure 4, we did not observe any RB in ARPE-19-nic cells, but RBBE was able to effectively penetrate into the cells and associated with mitochondria. Also, RBBE was able to stain evenly, whereas Mito-GFP was expressing at different levels in adjacent cells.

## Discussion

The present study demonstrates the effect of structural changes in a chemical species on cellular penetration. We speculated that masking hydrophilic functional groups may lead to enhanced cellular penetrance. As shown in this study, a classic example is the conversion of a carboxylic acid group into an ester derivative. In our case, RBBE did not de-esterify within 2 hours. Therefore, we assumed all the esters will not de-esterify within 2 hours, which makes quantification possible.

From a clinical perspective, treating patients with nondetrimental yet effective dosage for long-term treatments is crucial. It is assumed that patients may have less adverse effects when drugs are administered at subtherapeutic amounts to generate therapeutic amounts over time. To achieve this goal, chemical substances should accumulate and retain in the cells. Many researchers have found that making lipophilic versions of therapeutic agents increases cellular penetration.^9^^,^^24^ Presented retention data demonstrate cell impermeable rhodamine B could stay inside the cells after delivering it to the cell as a prodrug. However, it should be noted that structural aspects in the chemical substances may have a role in retention in the cells. RB has a fluorophore unit constructed from aromatic units. These aromatic units are known to make interactions with other substances containing π electrons to form π stacks.^25^^–^^27^ Also, despite the common belief that diffusion comprises the major force of getting chemicals into the cells,^28^ we did not observe similar amounts of dyes in the intracellular compartment after treating cells with similar concentrations of the dyes. Comparable fluorescence readings were obtained for ARPE-19-nic cells treated with RB esters, while parent dye RB was shown to be cell impermeable at 25 nM. We observed that RB esters enter the cells effectively, accumulate over time when administered with low concentrations (<100 nM) and retained the signal even up to 1 week after single exposure (25 nM). Moreover, even after incubating for 24 hours, RB was not able to penetrate into the cells suggesting in vitro cellular penetrance is time independent.

Delivering drugs into cells does not necessarily ensure in vivo efficacy because it is not directed toward the therapeutic target.^29^ Over the recent years, many researchers have found evidences of direct correlation between age-related diseases (e.g., AMD) and mitochondria. This makes mitochondria, the energy-producing subcellular organelle, an interesting therapeutic target. Owing to the inherent positive charge localized on the nitrogen atom in RB, the dye has been established as a mitochondrial stain. However, mitochondrial staining is achieved by using relatively high concentrations or formulated to increase staining efficiencies, while commonly used mitochondrial staining dyes are used at nanomolar concentrations (20–200 nM).^30^^–^^32^ Therefore, these ester derivatives will be an advantage, because they can be taken up by the RPE cells at very low concentrations.

Although these data suggest the possibility of successful delivery of chemical species into RPE cells and furthermore into mitochondria, we think it is necessary to replicate similar results using different chemical constructs to generalize our findings. If the concept used in our study is true for a wide variety of compounds, it will be beneficial for future ophthalmic medicines.

## Conclusions

Conversion of compounds containing hydrophilic carboxylic functional groups into ester constructs can enhance cellular penetration. With increasing evidence of mitochondria driving AMD pathology, we have shown that positively charged species with carboxylic acid functionality can be directed toward mitochondria via esterification. Observed results could be used as a guideline for ophthalmic drug discovery.

## Supplementary Material

Supplement 1
