# Supplementary material for: Investigating the Effect of Esterification on Retinal Pigment Epithelial Uptake Using Rhodamine B Derivatives
Source: Transl Vis Sci Technol. 2020 May 19;9(6):18. doi: 10.1167/tvst.9.6.18 (PMC7409196; doi:10.1167/tvst.9.6.18)
Supplement: Supplement 1 [file tvst-9-6-18_s001.pdf]

Supporting Information

Investigating the Effect of Esterification on Retinal Pigment Epithelial Uptake using Rhodamine B Derivatives

Chandima Bulumulla[1], Ruvanthi N. Kularatne[1], Timothy Catchpole[1], Alison Takacs[1], Abigail Christie[1], Alexa Gilfoyle[1], Timothy D. Nguyen[1], Mihaela C. Stefan[2,3], Karl G. Csaky[1,4]

[1]Retina Foundation of the Southwest, Dallas, Texas, 75231
[2]Department of Chemistry and Biochemistry, [3]Department of Bioengineering, The University of Texas at Dallas, Richardson, Texas, 75080
[4]Department of Ophthalmology, University of Texas Southwestern Medical Center, Dallas, Texas, 75390

Contents

1. NMR spectra of the dyes S3-S4

2. Absorption and emission spectra of the dyes S5

3. Morphology of ARPE-19-nic cells treated with dyes S5

3. Dose response data of the dyes S6

4. Co-localization CLSM images of ARPE-19-nic cells treated with dyes S6

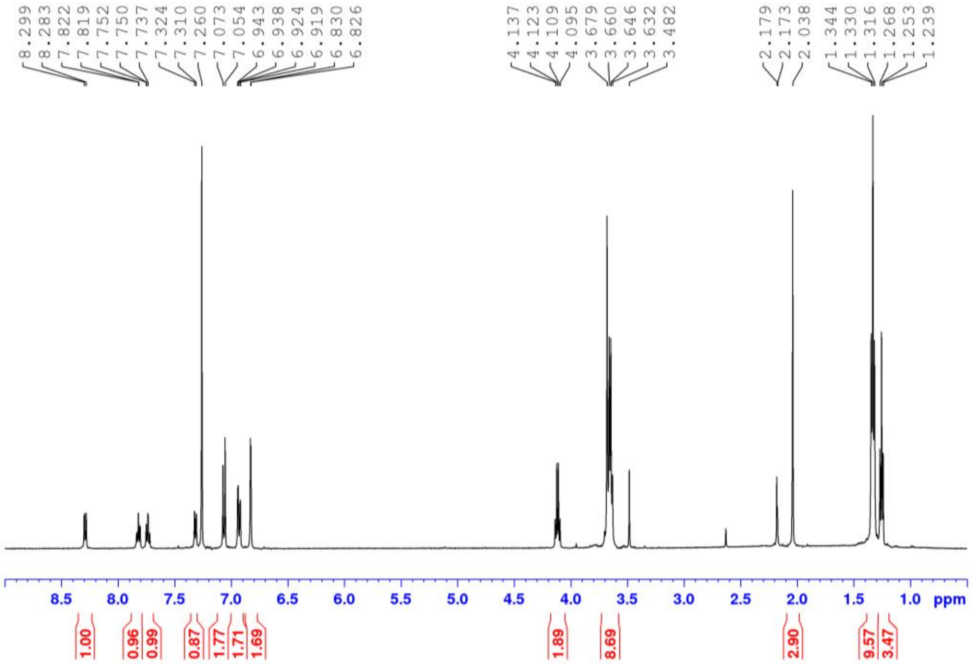

Figure S1. ^{1}H NMR spectrum of RBME in $CDCl_3$

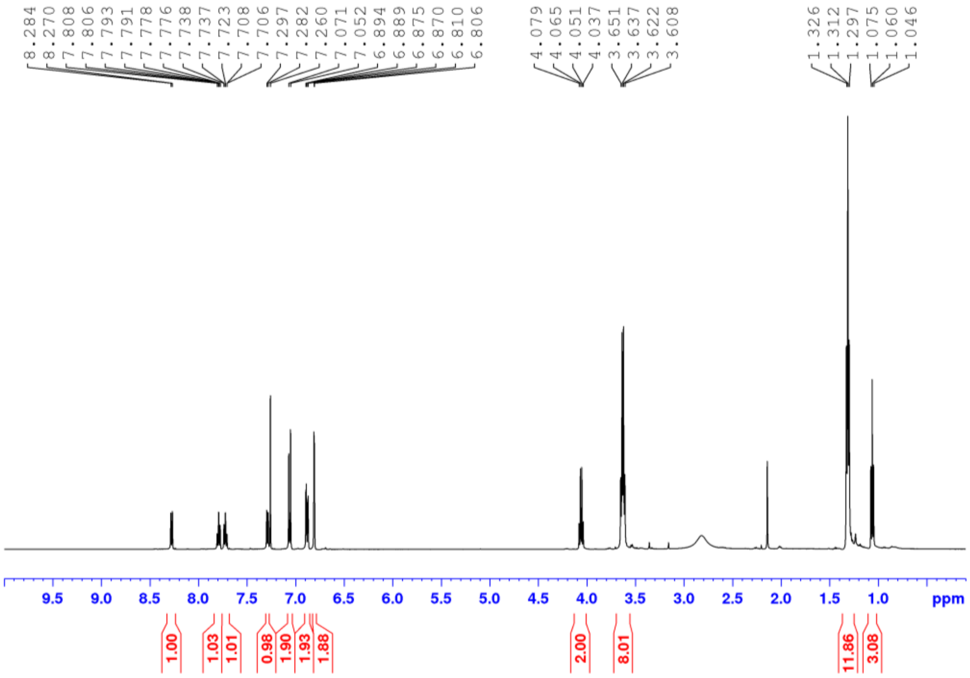

Figure S2. ^{1}H NMR spectrum of RBEE in $CDCl_3$

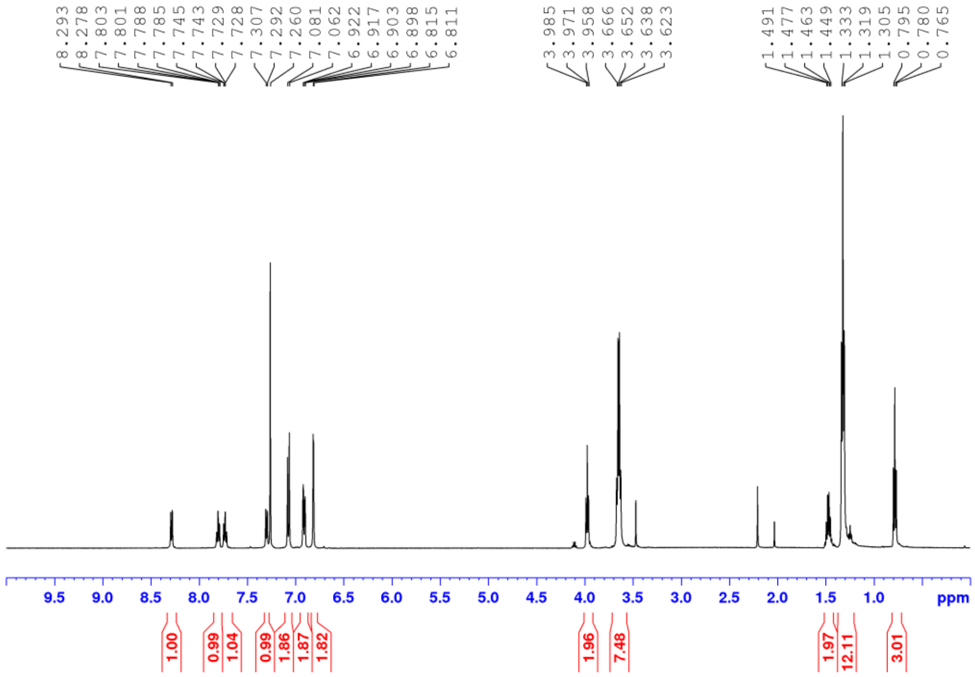

Figure S3. ^{1}H NMR spectrum of RBPE in $CDCl_3$

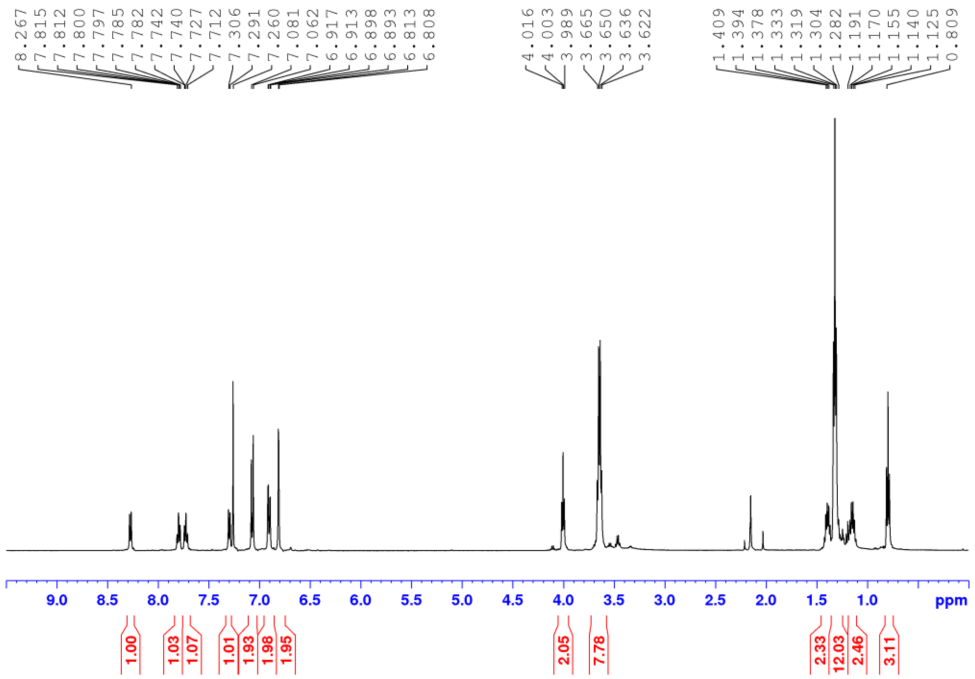

Figure S4. ^{1}H NMR spectrum of RBBE in $CDCl_3$

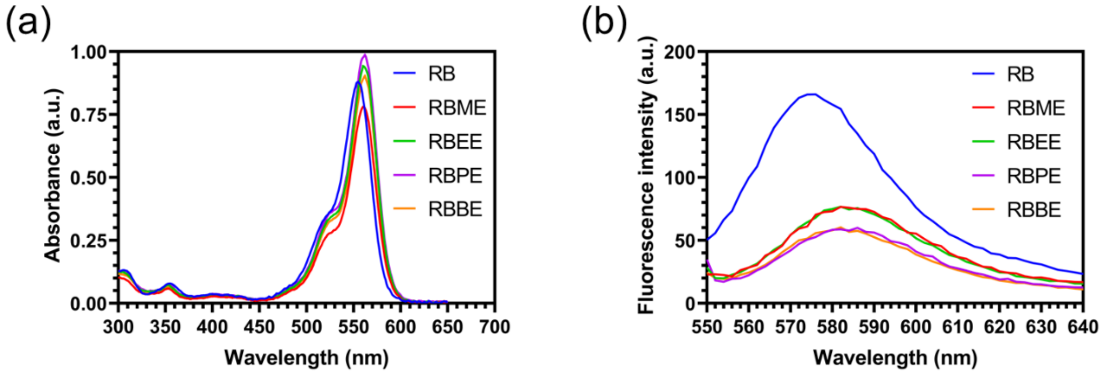

Figure S5. (a) Absorption, and (b) emission spectra of RB and ester derivatives in PBS

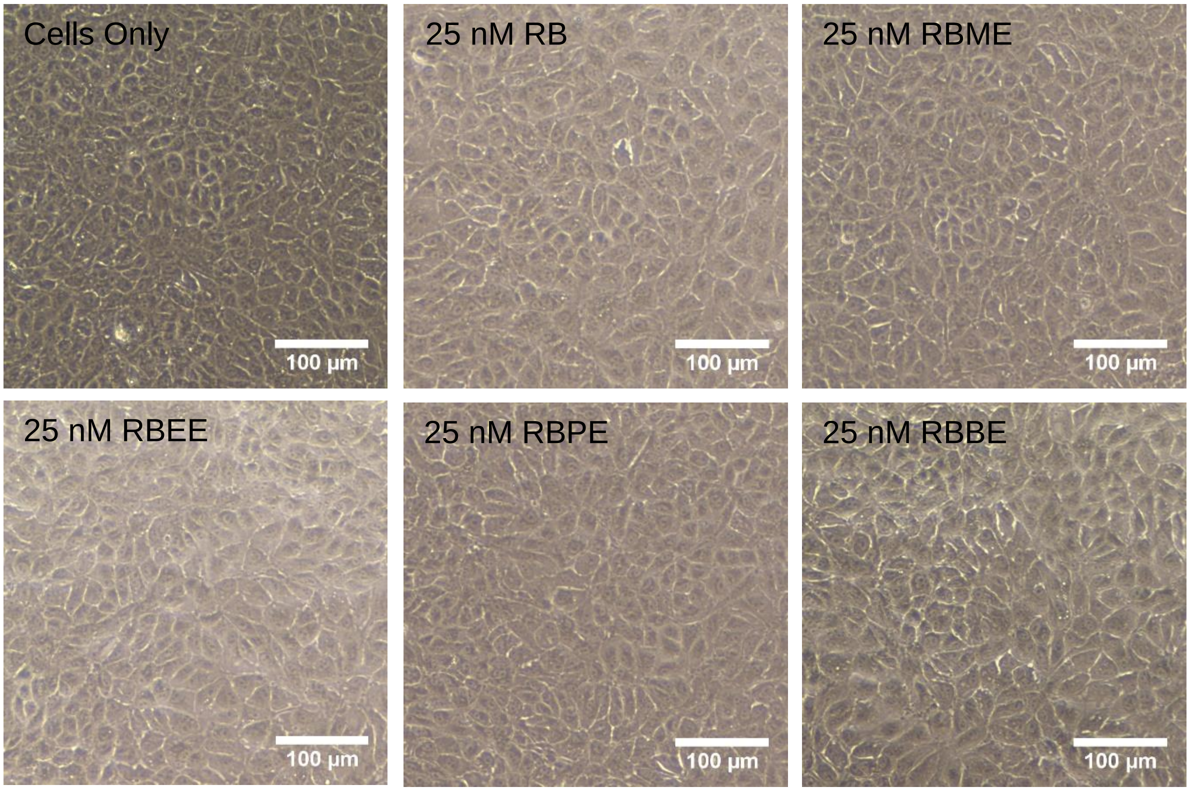

Figure S6. Bright-field images of ARPE-19-nic cells treated with dyes for 2 hours

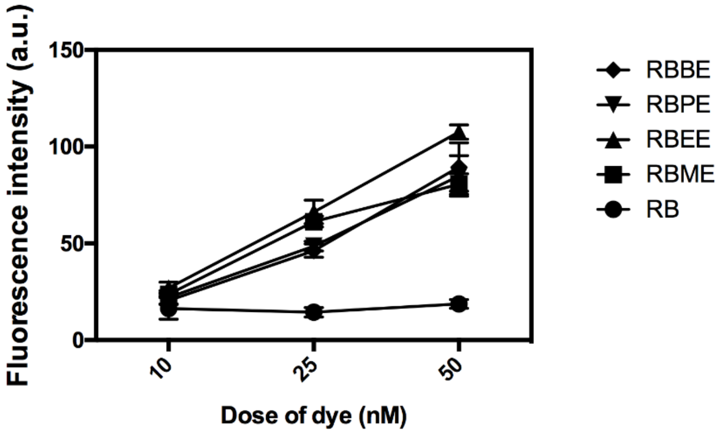

Figure S7. Dose response of the dyes treated at different concentrations in growth media for 2 hours

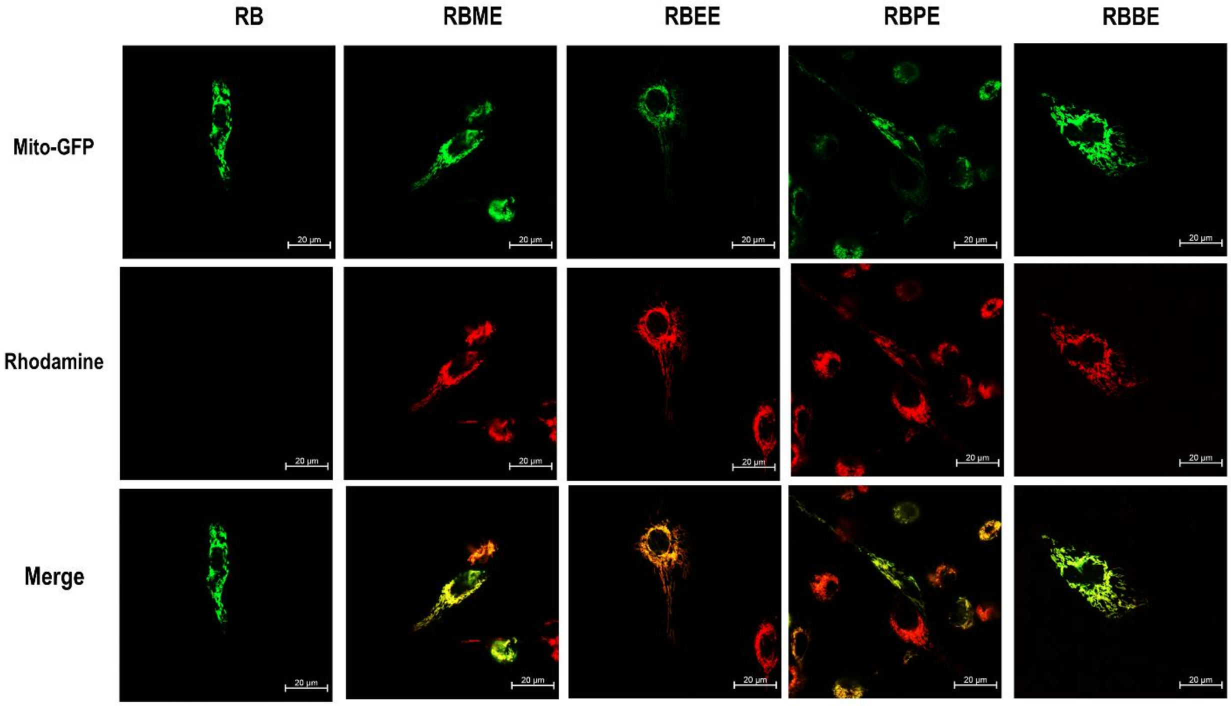

Figure S8. Co-localization CLSM images of ARPE-19-nic cells treated with 25 nM dye solutions for 30 minutes
